# Explosive Weapons Trauma Care Collective (EXTRACCT) Blast Injury Clinical Practice Guideline: Ocular Trauma

**DOI:** 10.1002/wjs.70204

**Published:** 2025-12-26

**Authors:** Emma Butterfield, Alistair Bolt, Gerry Clare, John Mattia, Aung Maw Tin‐U, Iddi Ndyabawe, Larry Schwab, Siegfried Karl Wagner, the EXTRACCT Clinical Practice Guidelines Collaboration

**Affiliations:** ^1^ The Royal London Hospital Barts Health NHS Trust London UK; ^2^ East Anglian Air Ambulance Norwich UK; ^3^ Formerly Pharmacist Norfolk and Norwich University Hospital Norwich UK; ^4^ NIHR Moorfields Biomedical Research Centre London UK; ^5^ Lowell and Ruth Gess UMC Eye Hospital Freetown Sierra Leone; ^6^ Department of Ophthalmology St Bernard's Hospital Hanwell Gibraltar; ^7^ Department of Ophthalmology Kisubi Hospital Entebbe Uganda; ^8^ School of Medicine West Virginia University Morgantown West Virginia USA; ^9^ Institute of Ophthalmology University College London London UK

**Keywords:** endophthalmitis, globe rupture, hyphema, keratitis, lid laceration, ocular trauma

## Abstract

**Introduction:**

This clinical practice guideline from the Explosive Weapons Trauma Care Collective (EXTRACCT) group reviews current best practice for the management of ocular trauma in conflict‐affected regions, where explosive weapons are used and healthcare infrastructure is limited.

**Methods:**

An expert literature review of current practice is presented with practical resource‐adapted guidelines constructed through expert consensus from ophthalmologists, emergency care providers and allied health professionals with field experience.

**Results:**

The guideline provides recommendations for the assessment, classification and management of major and minor ocular injuries encountered in low‐resource settings, particularly during conflict. Guidance is written for frontline healthcare workers who may be addressing such injuries in the absence of specialist ophthalmology expertise and equipment. Dosing of ophthalmic therapeutics is provided.

**Conclusion:**

Actionable context‐appropriate strategies to manage ocular trauma caused by explosive weapons can reduce vision loss and improve patient outcomes where specialized ophthalmic care is scarce.

## Graphical abstract

This clinical practice guideline from the Explosive Weapons Trauma Care Collective (EXTRACCT) group reviews current best practice for the management of ocular trauma in conflict‐affected regions, where explosive weapons are used and healthcare infrastructure is limited. An expert literature review of current practice is presented with practical resource‐adapted guidelines constructed through expert consensus from ophthalmologists, emergency care providers and allied health professionals with field experience.

## 1 Objectives

This clinical practice guideline (CPG) provides guidance on how to assess and treat eye injuries in a limited‐resource context, where an ophthalmologist may not be available.

It aims to advise on assessment of an eye injury; which eye injuries to refer to an ophthalmologist; the immediate interventions that should be performed before the patient is transferred; treatment if there is no possibility of referring to an ophthalmologist and the diagnosis and treatment of the common complications of eye injury (preseptal and orbital cellulitis, infectious keratitis, acute angle closure glaucoma). It is not a substitute for clinical judgment, and where expert advice or a higher level of care is available, it should be sought.

Outcomes of eye injuries which do not receive specialist ophthalmic care are much worse: all eye injuries benefit from expert treatment. The sooner patients are transferred, the better their outcomes.

## 2 Background

Ocular trauma predominantly affects young males (4:1 to 2:1 male:female ratio) and is associated with socioeconomic deprivation [1, 2, 3, 4, 5, 6]. Farm work is a common cause of injury, particularly injuries by sticks or stones [1, 2, 5]. Children are more likely to be injured during play [2, 7] and infants by self‐injury [8]. Epidemiology varies between and within countries: workplace injuries are commoner in rural areas and where eye protection is not mandatory [9]. Blunt injuries are the commonest cause of eye injury globally. About 20% of injuries are due to assault, although this is higher in some contexts [2, 9], and gender differences have been reported in some contexts, with a report of elderly women predominantly suffering ocular trauma due to assault [1]. In modern conflicts, the eye is disproportionately affected by trauma [9].

Expert treatment can be sight‐saving, even in severely injured eyes, and all patients with eye injuries with a change in vision should be assessed by an ophthalmologist, where one is available.

### 2.1 Guideline Development and Review Methods

The CPG was structured and developed in accordance with the Explosive Weapons Trauma Care Collective (EXTRACCT) Clinical Practice Guideline Development Process (Supplementary Material). EXTRACCT aims to develop CPGs tailored to caring for civilian blast injury victims in low‐resource settings. Targeted at diverse medical personnel, this CPG aims to standardize care, improve outcomes and fill knowledge gaps. Development was led by experts experienced in resource‐constrained trauma care and informed by targeted literature reviews.

A literature search with the term “ocular trauma” and the MeSH Terms “Developing Country” or “Resource‐Limited Setting” was conducted in the MEDLINE database through PubMed from inception to 6/6/2025. 11 articles were located, of which on title and abstract review, 8 were relevant. Full text was obtained for all relevant articles. Gray literature was hand‐searched. Guidelines were generally specific to an injury type (e.g., mustard gas [10], mechanical trauma [11]); one ocular trauma guideline focused on recommendations in light of the COVID‐19 pandemic [12] and another on care provided by US military personnel [13]. All assumed the availability of an ophthalmologist. Where there were no published recommendations on how extant treatment guidelines should be modified in a low‐resource setting, consensus was reached by the authors after discussion.

### 2.2 Patient Evaluation

**FIGURE 1:**
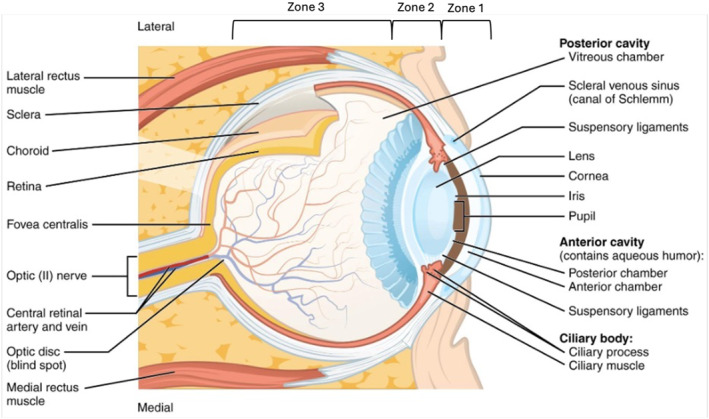
Anatomy of the eye. Note that Zone 1 includes the cornea and Zone 2 the anterior sclera extending from the limbus to rectus muscle insertions. Zone 3 encompasses the sclera posterior to the rectus insertions. Illustration modified from Anatomy & Physiology, Connexions Web site http://cnx.org/content/col11496/1.6/. Licensed under Creative Commons license.

**FIGURE 2:**
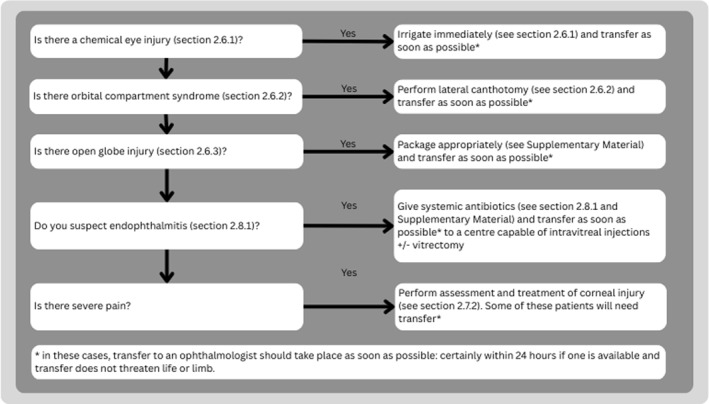
Patient evaluation in ocular trauma.

Examination of the eye should proceed only after a complete primary survey with identification and treatment of life‐ or limb‐threatening injuries. Clinicians should be familiar with the anatomy of the eye (Figure 1) and extraocular structures [13]. A systematic approach to the assessment of a patient with ocular trauma is given in Figure 2. Acute visual loss is disorienting and frightening: explain to the patient what you are doing [9]. If there is a chemical injury to the eye, irrigate the eye (see chemical eye injury) *before* examining the eye [9]. Patients with severe eye injuries should be admitted and examined twice daily, or more often if symptoms change. Examine outpatient cases daily.

### 2.3 General Treatment

Tetanus prophylaxis should be provided to any patient with a penetrating wound who may not have completed their tetanus immunization. They should be given systemic or topical antibiotics where indicated (Supplementary Material) and adequate analgesia. Vomiting increases intraocular pressure and can worsen eye injuries: treat all patients with regular anti‐emetics (ondansetron 4–8 mg three times daily PO/IV is preferable to metoclopramide 10 mg three times daily PO/IV) [9].

### 2.4 Description of the Injury

To document the injury and receive specialist advice, it is essential to accurately describe the following.

- Mechanism of injury—be aware of the risk of open globe injuries and intraocular foreign bodies in injuries caused by grinding, metal cutting or explosions
- Integrity of the globe—eye injuries are classified according to whether the globe is closed (the eyeball in intact) or open (the wall of the eye has been penetrated). Closed globe injuries can be additionally divided into contusions (blunt injury with no wound) or lamellar lacerations (partial thickness injury). Open globe injuries can be divided into ruptures (full thickness injury with a blunt object) or lacerations (full thickness injury with a sharp object). Lacerations typically require surgical treatment and can be further subdivided as penetrating (single entry wound) or perforating (entry and exit wound present), both of which may involve an intraocular foreign body (retained foreign object).
- Severity of injury—describe visual acuity and the presence of a relative afferent pupillary defect
- Zone of injury—identify which structures of the eye have been injured, and whether the injury has perforated them.
  - Zone 1 injuries involve just the cornea
  - Zone 2 injuries involve the anterior 5–7 mm of the sclera (anterior to the rectus muscle insertions)
  - Zone 3 injuries involve the posterior sclera

### 2.5 Triage and Immediate Management

**FIGURE 3:**
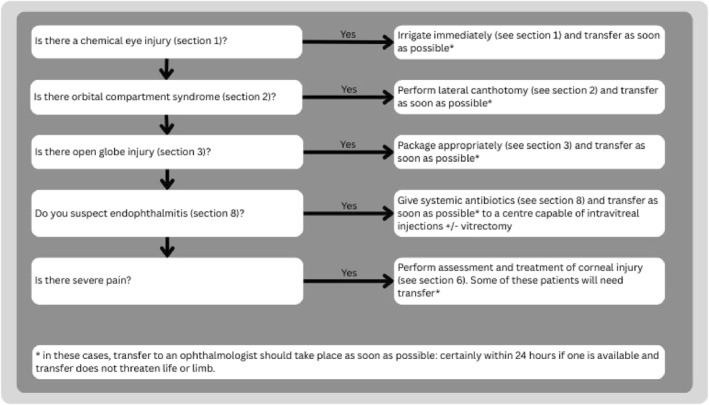
Sequence of immediate treatment priorities in ocular trauma.

Treat all life‐ and/or limb‐threatening injuries before assessing the eye injury. Then address sight‐threatening injuries in a sequential order prioritizing severity (Figure 3).

### 2.6 Sight‐Threatening Emergencies

All sight‐threatening emergencies should be referred to an ophthalmologist, if one is available.

#### 2.6.1 Chemical Eye Injury

A chemical eye injury is an injury to the cornea, conjunctiva and/or lid caused by acid or alkali. Patients typically report a painful eye, possibly with reduced visual acuity, after exposure to chemical liquids or powders (e.g., bleach). The goals of care are to remove the chemical which has caused the injury and prevent subsequent infection.

Only irrigation of the eye can stop further injury. Irrigate before examination, unless there is an open globe, in which case irrigation is contraindicated. You may apply a local anesthetic eye drop (e.g., tetracaine) for analgesia prior to irrigation. Note that 1% lidocaine for IV administration has a pH of 6 and is therefore not recommended for use as an eyedrop [11].

For irrigation of the eye, use drinking water or 0.9% sodium chloride or a physiological electrolyte solution (e.g., Ringer's). If IV fluids, you can irrigate the eyes by connecting IV tubing to clean nasal oxygen cannula and positioning the cannula on the bridge of the nose, so each cannula is resting at the inner corner of the eye.

Ensure all foreign bodies are removed from under the eyelids, by irrigating with the lid everted and/or using a cotton bud. Continue irrigation until the pH (tested with litmus or universal indicator paper under the eyelid, in the conjunctival fornix) is normal, then examine the eye. If pH testing is not available, use a minimum of 3 L of fluid [12].

Further treatment includes topical antibiotics (see Supplementary Material), topical cycloplegics for analgesia (cyclopentolate 1% three times a day for 1 week), oral analgesia (e.g., paracetamol/ibuprofen) and sterile artificial tears.

Specialist treatment may be required. Following discussion with an ophthalmologist, some patients may benefit from topical steroid eye drops (0.1% dexamethasone drops) at least four times a day. These are immunosuppressant: it is essential the patient does not apply any non‐prescribed or traditional ointments to the eye and washes their hands with soap before applying their medication. For severe injuries (especially alkaline), the following may be considered: doxycycline 200 mg PO once daily or tetracycline 250 mg PO four times a day; topical sodium citrate 10% or potassium/sodium ascorbate 10% eye drops hourly where available; PO sodium ascorbate (ascorbic acid, vitamin C) 2 g PO four times a day. Patients may benefit from antiglaucoma medications (timolol 0.5% twice a day) if the intraocular pressure (IOP) is high.

#### 2.6.2 Orbital Compartment Syndrome

Bleeding between the eyeball and the extraocular muscles may compress the retinal vessels and cause ischemia (i.e., orbital compartment syndrome): this can result in permanent sight loss within 90 min of injury [14].

Orbital compartment syndrome (OCS) should be suspected in blunt injuries to the orbit: conscious patients with OCS may report eye pain, reduced visual acuity and/or loss of color sensitivity; clinical examination may reveal a relative afferent pupillary defect. The eye is typically proptosed and tense to touch as IOP is raised. The goal of care is to release the pressure in the orbital compartment by performing lateral canthotomy: this is a sight‐saving procedure which can be performed safely by non‐ophthalmologists and the area heals well. The procedure is well‐described [15] and illustrated in open‐access resources [16], and requires only mosquito forceps, Westcott or iris scissors (with 3–4 cm blades), and local anesthetic for the conscious patient.

- Anesthetize the skin lateral to the eye with 1% lidocaine +/− 1:200,000 adrenaline/epinephrine.
- Insert the forceps into the lateral canthal fold and clamp the skin for 1 min to reduce bleeding, then remove the forceps.
- Insert the scissors into the lateral canthal fold and make a 1 cm incision laterally toward the orbital rim along the line where you clamped.
- Use the forceps to retract the inferior eyelid, exposing the inferior lateral canthal tendon; cut through it with the scissors.
- If the procedure is successful, you will see the eyeball visually recess into the orbit; if you do not, proceed to cut the superior limb of the lateral canthal tendon.
- If you cannot visualize the canthal tendons, you may be able to palpate them with the scissors: they should feel tense and taut like a “guitar string”.

After lateral canthotomy, check pupillary reflexes and visual acuity, check IOP if available, start prophylactic antibiotics (Supplementary Material), and refer the patient urgently to an ophthalmologist. If lateral canthotomy cannot be performed locally, give acetazolamide 500 mg PO/IV as a single dose, shield the eye (see Supplementary Material) and refer the patient immediately to a facility where it can be performed.

#### 2.6.3 Open Globe Injury (including Intraocular Foreign Bodies)

Injuries involving a full‐thickness defect of the eye wall are called “open globe” injuries. They may be caused by sharp (penetrating or perforating) injuries to the globe or by blunt injuries causing globe rupture.

Penetrating or perforating injuries may be associated with an intraocular foreign body. Globe rupture most often occurs posteriorly, behind the rectus muscle insertions, so no anterior wound may be visible [10]. Loss of the iris or lens, or a “step” in the conjunctiva are pathognomonic of rupture (1).

Suspect an open globe injury in all eyelid lacerations, or following any injury where visual acuity is reduced and/or the red reflex is impaired. Open globe injuries may be difficult to detect, but a relative afferent pupillary defect is common and there is usually hyphaema. Intraocular pressure may be low (“collapsed eyeball”) or conversely may be raised if presentation is delayed. Suspect intraocular foreign bodies in any case of penetrating eye injury. The goal of care is to identify the possibility of an open globe injury, prevent secondary injury or infection and refer to an ophthalmologist.

If open globe injury is suspected, determine the severity of injury (examine the eye and measure visual acuity) and prevent further injury by applying an appropriate, rigid, eye shield. Prescribe anti‐emetics to reduce exacerbation of rupture from emesis. Give IV/PO antibiotics (Supplementary Material) and discuss topical antibiotics with an ophthalmologist. Refer to an ophthalmologist immediately.

Irrigation is contraindicated and foreign bodies or prolapsed tissues should not be removed. Foreign bodies are difficult to detect: 30% of intraocular metallic foreign bodies are not detected by x‐ray: MRI is absolutely contraindicated if there is a suspected metallic foreign body. The use of magnets to remove intraocular foreign bodies should be reserved for ophthalmologists.

Where an ophthalmologist is not available, manage conservatively: do not attempt surgical intervention, consider giving systemic broad spectrum antibiotics (e.g., fluoroquinolones—Supplementary Material) and protect the eye with a rigid shield (Supplementary Material).

Even with the best care, blindness following open globe injury is common.

#### 2.6.4 Closed Globe Injury

A closed globe injury is an eye injury in which there is damage to the globe (e.g., hyphema, vitreous hemorrhage or retinal detachment) without a full thickness wound to the eye wall. It should be suspected if there is blunt trauma to the eye with acute visual loss, and no signs of open globe injury. Suspect retinal detachment where there is a defect in the visual field. The goals of care are to maintain normal intraocular pressure (thus avoiding preventable visual loss) and to avoid rebleeding into the anterior or posterior segments.

Essential treatment includes assessment of the injury, detection of raised intraocular pressure and rebleeding and prevention and treatment of raised intraocular pressure as needed.

For assessment, evaluate the severity of hyphema (i.e., how much of the pupil is covered) and the red reflex. Loss of the red reflex in closed globe injury may indicate severe (”8‐ball” or “black ball”) hyphema, vitreous hemorrhage or retinal detachment.

To prevent raised IOP, raise the head of the bed 45°. Give cycloplegic eye drops (cyclopentolate 1% three times a day or atropine 1% three times a day) and if there is no corneal injury, give prednisolone 1% eye drops four times a day (if in doubt about a corneal injury, do not give). Minimize physical activity as much as possible.

You should assess rebleeding regularly through VA and IOP daily (particularly at days 3–5 when clot retraction can cause rebleeding). Avoid NSAIDS which can increase bleeding risk.

To treat raised IOP (suspected either by IOP measurement, or because the patient complains of increasing pain, reduced VA and develops a RAPD), give timolol eye drops 0.5% twice a day (contraindication: asthma, heart block) and acetazolamide 500 mg PO as a single dose, followed by 250 mg PO four times a day (contraindicated in patients with sickle cell disease or trait) or 3% hypertonic saline 250 mL IV or mannitol: 1 g/kg IV over 30–60 min, or oral glycerol/glycerine 1.5 g/kg (=3 mL/kg of 50% solution) as a single dose, diluted with water or fruit juice.

If the hyphema covers the pupil and IOP is raised, early evacuation (by an ophthalmologist) is indicated. If IOP rises, re‐discuss with an ophthalmologist.

### 2.7 Non‐Sight Threatening Injuries

These injuries should be referred to an ophthalmologist if indicated below.

#### 2.7.1 Eyelid Laceration

These include any cut or tear to the tissues of the eyelid and are clinically obvious. Any eyelid laceration which involves any of the following is defined as a complicated laceration and should be referred to an ophthalmologist.

- full‐thickness or through the lid margin
- associated with tissue loss or orbital fat prolapse
- involves the lacrimal ducts, the levator aponeurosis, the superior rectus muscle or the medial canthal tendon
- associated with a globe injury or intraocular foreign body

In all eyelid lacerations, the first priority is to determine if there is an associated open or closed globe injury before arranging appropriate first aid and referral for the laceration.

In simple eyelid lacerations, the risk of infection is low unless the injury is caused by a bite, or there is visible fat (indicating penetration of the orbital septum, a key physical barrier to infection, see Supplementary Material). Do not attempt to excise or suture orbital tissue prior to referral: it can cause bleeding which threatens sight. Irrigate the wound very gently with 0.9% sodium chloride unless you suspect open globe injury (where irrigation is contraindicated). Gently close the eyelid wound with sticking plaster or cover exposed tissues very lightly with clingfilm to prevent them drying out, before applying a rigid eye shield and referring the patient to an ophthalmologist.

Expert closure (by an ophthalmologist) is usually required to preserve lid function (which is necessary to prevent subsequent corneal injury and painful blindness).

#### 2.7.2 Corneal Foreign Bodies and Abrasions

Corneal foreign body is a particle which is lodged in (but has not penetrated through) the cornea. A corneal abrasion is a non‐penetrating corneal injury. Foreign bodies are immediately recognised by the conscious patient, who will describe an acute onset of foreign body sensation and corneal pain.

In unconscious patients, perform a fluorescein exam. The goals of care are to determine if there is an associated open globe injury, which should be prioritized and prevent secondary infection.

**FIGURE 4:**
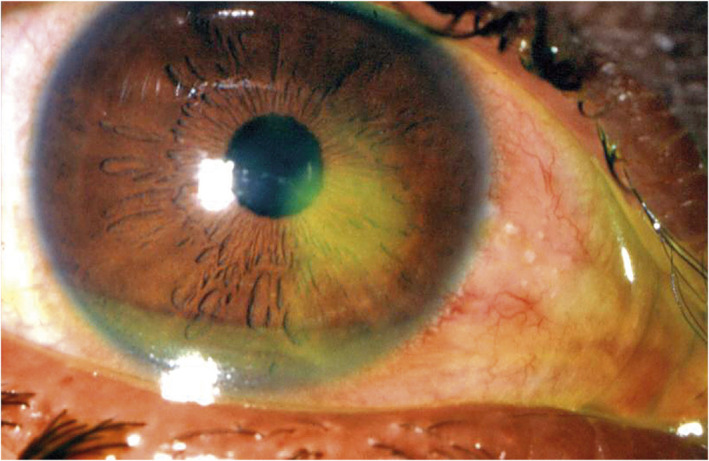
Acute corneal abrasion Photo: Karin Lecuona/Dept. of Ophthalmology University of Cape Town. Published in: Community Eye Health Journal Vol. 18 No. 55 OCTOBER 2005 www.cehjournal.org. Licensed under Creative Commons.

**FIGURE 5:**
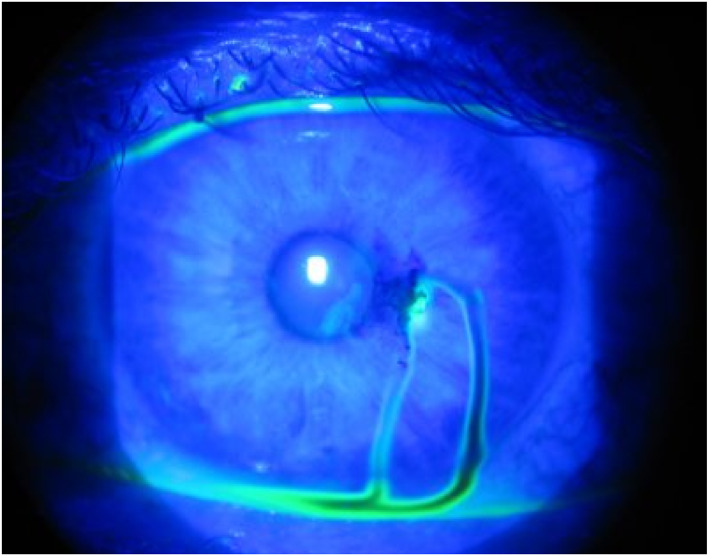
Positive Seidel test. Photo: Jordan M. Graff, MD/University of Iowa. Published in: Ophthalmic Atlas Images by EyeRounds.org, The University of Iowa. Licensed under Creative Commons.

Following your routine ophthalmic exam, apply a topical anesthetic drop to permit examination of the injured eye. Stain the cornea with fluorescein (2% or less) to identify acute corneal abrasions (Figure 4), which are not visible to the naked eye. A dark streak passing through the fluorescein indicates aqueous humor leaking onto the surface of the eye (Seidel test: Figure 5) through a penetrating wound in the cornea, and is a sign of an open globe injury (see Section 1). If there is an open globe injury, do not evert the eyelids or attempt to remove any foreign bodies; treat as for open globe injury. If you are confident there is no globe injury, evert the upper and lower eyelids to identify foreign bodies in the fornices. Remove superficial foreign bodies with a soft cotton bud or piece of damp gauze [9]; embedded foreign bodies may be removed by scraping the cornea with a needle. Treat the injured conjunctiva with regular topical antibiotics to promote healing (see Supplementary Material).

#### 2.7.3 Orbital Fracture

A break in any of the bones of the eye socket constitutes an orbital fracture. Following blunt trauma, suspect an orbital fracture in any patient with a “step” in the orbital rim on palpation, numbness of the skin below the eye (infraorbital nerve injury), double vision or pain on eye movement (trapping of the extraocular muscles) or if the globe appears sunken back into the orbit (enophthalmos) or there is trismus or malocclusion of the teeth (suggesting injury to the zygomatic‐maxillary bones). The goals of care are to identify any associated globe injury (see above) and to prevent and treat further injury.

For essential treatment, give ice packs hourly to reduce swelling in the first 24 h. If there are signs of orbital compartment syndrome (see above), perform lateral canthotomy. To prevent raised IOP, raise the head of the bed 45°. Do not allow the patient to blow their nose (which can cause air to enter the orbit) and give nasal decongestants: oxymetazoline nasal spray twice a day or pseudoephedrine 30 mg PO four times a day. Give for only 3 days to prevent rebound rhinorrhea. To prevent secondary infection, give appropriate antibiotics (Supplementary Material).

Entrapment of the extraocular muscles may cause bradyarrhythmia and vomiting, due to vagal stimulation via the oculocardiac reflex: Treat with anticholinergics (glycopyrronium four to five mcg/kg IV in adults and children or atropine 20 mcg/kg IV in children, 0.5–1.2 mg in adults; repeat doses will be necessary). If present, give extra antiemetics and refer for urgent surgical repair.

### 2.8 Complications of Eye Injury

#### 2.8.1 Infections

Cellulitis in and around the eye must be accurately diagnosed and classified to ensure appropriate treatment. Both preseptal and orbital cellulitis present with painful swelling and erythema of the eyelid and may have an associated abscess (Table 1).

**TABLE 1:** Distinguishing features between preseptal and orbital cellulitis.

Preseptal cellulitis	Orbital cellulitis
Infection of the skin and soft tissues of the eyelid, anterior to the orbital septum (which runs from the bony orbit to the tarsal plate of the eyelid ‐ see Figure 6).	Infection of the orbit, posterior to the orbital septum (Figure 6).
Visual acuity is normal	Visual acuity may be reduced
Pupillary light responses are normal	Pupillary responses reduced with a relative afferent pupillary defect
Eye movements painless with a full range of motion	Eye movements are painful and limited.

**FIGURE 6:**
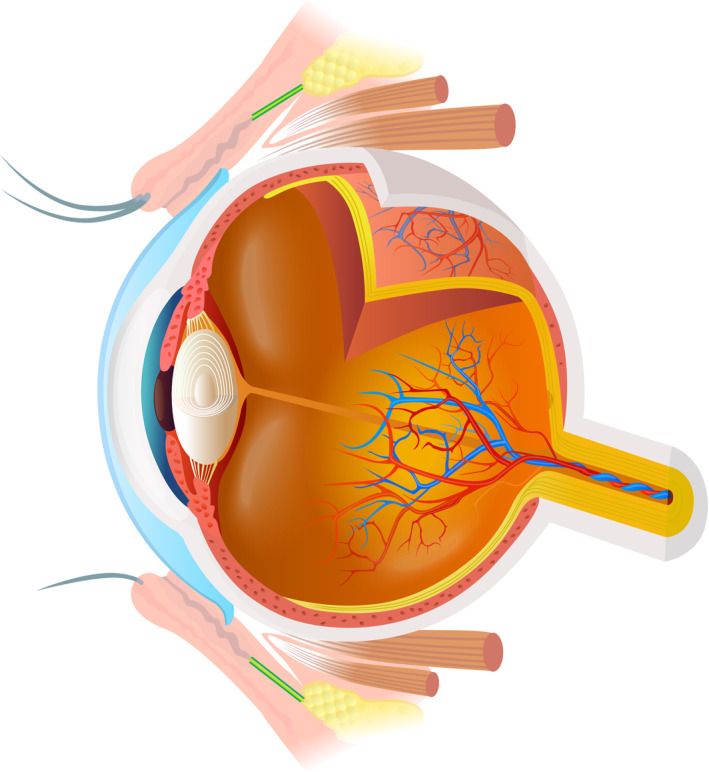
Sagittal section through the eye, showing the orbital septum (green) attached to the bony orbit and the tarsal plate of the eyelid, forming an anatomical barrier. Image credit: iStock.com/socotra.

Any abscess on the eyelid must be incised and drained with utmost care to avoid spread of infection posteriorly through the orbital septum. Both preseptal and orbital cellulitis are treated with systemic antibiotics (see Supplementary Material); if you have any doubt about the diagnosis, or if the patient is under 5 years old, treat for orbital cellulitis. Any loss of visual acuity, relative afferent pupillary defect, or pain on eye movement, should lead the clinician to suspect and treat for orbital cellulitis.

Patients with post‐traumatic preseptal cellulitis should be managed as inpatients. If this is impossible, they should be reviewed daily and preferably managed by an ophthalmologist and multidisciplinary team in case of complications: refer if possible.

Symptoms in both eyes, after trauma to one eye, is highly suggestive of cavernous sinus thrombosis and mandates referral to a neurosurgical center if available.

Endophthalmitis is an infection of the globe of the eye, associated with purulent vitreous fluid and will only occur if the globe has been breached. Suspect endophthalmitis in patients with a history of (possible) open globe injury, who complain of blurred vision. Most patients with endophthalmitis have hypopyon on examination, 75% will experience pain [17, 18].

In all patients with cellulitis or endophthalmitis, check for clinical signs of meningism; if you suspect intracranial extension of infection, give antibiotics as for orbital cellulitis (including ceftriaxone 2g IV twice a day) and refer to the highest possible level of care. Patients who are clinically septic should be managed in accordance with international guidance [19].

#### 2.8.2 Infectious Keratitis

Infectious keratitis is an infection of the cornea which commonly occurs following untreated corneal abrasions. Patients report a delayed onset of eye redness, pain, photophobia and discharge following ocular trauma. In temperate zones, bacteria are the commonest cause, and polymicrobial infections are common. Fungal keratitis is more likely in tropical climates, more so in traumatic injuries caused by contact with vegetable matter. Acanthameba keratitis is more likely in contact lens wearers, especially following contact with contaminated water. The goals of care are to identify and treat the superficial infection and exclude infection in the anterior chamber.

Fluorescein examination in infectious keratitis shows areas of corneal epithelial damage. Examine carefully for signs of inflammation in the anterior chamber (pus) or symptoms of anterior uveitis (photophobia) to exclude deeper infection. Where deeper infection has been excluded, fluoroquinolone (e.g., moxifloxacin 0.5% or ciprofloxacin 0.3%) eye drops are the mainstay of treatment and should be given every 1 h around the clock (i.e., waking the patient at night to apply them) for the first 48 h, then every 1 h during the day until day 5, tapering thereafter according to clinical response. If there is a strong risk of fungal keratitis, add Natamycin 5% drops (same frequency as fluoroquinolone, alternating between the two treatments every half an hour). Give cycloplegic eye drop (cyclopentolate 1%, 1 drop three times a day) for photophobia.

#### 2.8.3 Acute Ocular Hypertension (Raised Intraocular Pressure)

Raised pressure within the eyeball (raised intraocular pressure) may occur following ocular trauma and cause retinal ischemia. Signs of raised IOP include eye pain, reduced visual acuity, pupil irregularity and/or a palpably firm eyeball). The risk is highest in elderly patients, or where there is injury to the lens or iris, hyphema or poor VA (< 20/200). Fifty‐two percent of patients with a total hyphema will develop raised IOP which necessitates surgery [9]. The goals of care are to identify and treat raised intraocular pressure; recognizing that untreated ocular hypertension threatens sight.

If you suspect raised IOP following injury, start medical management and refer to an ophthalmologist.

Topical treatment includes beta‐blockers (0.5% timolol twice a day ‐ avoid in asthma) [13]. Systemic treatments include acetazolamide 500 mg PO as a single dose, followed by 250 mg PO four times a day (contraindicated in patients with sickle cell disease or trait) or 3% hypertonic saline 250 mL IV or mannitol: 1 g/kg IV over 30–60 min, or oral glycerol/glycerine 1.5 g/kg (=3 mL/kg of 50% solution) as a single dose, diluted with water or fruit juice.

## 3 Conclusions

This CPG has provided frontline healthcare professionals with a structured approach to the evaluation, diagnosis and management options for ocular injuries from blast‐related trauma.

## Author Contributions

**Emma Butterfield:** conceptualization, methodology, writing – original draft, writing – review and editing, project administration. **Alistair Bolt:** writing – review and editing, formal analysis. **Gerry Clare:** writing – review and editing, formal analysis. **John Mattia:** writing – review and editing, formal analysis. **Aung Maw Tin‐U:** writing – review and editing, formal analysis. **Iddi Ndyabawe:** writing – review and editing, formal analysis. **Larry Schwab:** writing – review and editing, formal analysis. **Siegfried Karl Wagner:** conceptualization, methodology, writing – review and editing.

## Ethics Statement

The authors have nothing to report.

## Conflicts of Interest

The authors declare no conflicts of interest.

## Supporting information


Supporting Information S1
