# Supplementary material for: Explosive Weapons Trauma Care Collective (EXTRACCT) Blast Injury Clinical Practice Guideline: Ocular Trauma
Source: World J Surg. 2025 Dec 26;50(2):289–97. doi: 10.1002/wjs.70204 (PMC12904851; doi:10.1002/wjs.70204)
Supplement: Supplementary file 1 — Supporting Information S1 [file WJS-50-289-s001.docx]

# SUPPLEMENTARY MATERIAL

# Explosive Weapons Trauma Care Collective (EXTRACCT) Blast Injury Clinical Practice Guideline: Ocular Trauma

Emma Butterfield^1, 2^, Alistair Bolt^3^, Gerry Clare^4^, John Mattia^5^, Aung Maw Tin-U^6^, Iddie Ndyabawe^7^, Larry Schwab^8^, Siegfried Karl Wagner*^4,9^

On behalf of the EXTRACCT Clinical Practice Guidelines Collaboration

**^*^Correspondence and reprint requests:** Siegfried K Wagner, NIHR Moorfields Biomedical Research Centre, London, United Kingdom. Tel: +44 207 253 3411 Email: [s.wagner@ucl.ac.uk](mailto:s.wagner@ucl.ac.uk)

##

###

### Antibiotic treatment

Doses given are for adults and should be adjusted appropriately in children according to your national formulary and guidelines. The British National Formulary for Children is a good reference.

| Pathology | Antibiotic options | Duration |
| --- | --- | --- |
| Chemical eye injury and corneal abrasions | Topical chloramphenicol 0.5% (drop or ointment) four times a day | One week |
| Orbital compartment syndrome (following lateral canthotomy) | Give one of   - Amoxicillin/clavulanic acid 500/125 mg PO three times a day - *or* Moxifloxacin 400 mg PO once a day - *or* Levofloxacin 750 mg PO once a day - *or* Ertapenem 1g IV/IO once a day (4) | One week |
| Eyelid laceration due to animal/human bite, or with visible fat | Give one of   - Moxifloxacin 400 mg PO once a day - *or* Levofloxacin 750 mg PO once a day - *or* Amoxicillin/clavulanic acid 500/125 mg PO three times a day - *or* Ertapenem 1g IV/IO once a day | One week  Consider rabies and tetanus post-exposure vaccination. |
| Conjunctival injuries/corneal foreign bodies with persistent abrasion | Give one of:   - Topical tetracycline 1% 6 times a day - *or* Topical chloramphenicol 1% 6 times a day | One week |
| Penetrating globe injury (or suspected penetrating globe injury) | Give one of:   - Moxifloxacin 400 mg PO once a day - *or* Levofloxacin 750 mg PO once a day | Until review by an ophthalmologist |
| Preseptal cellulitis | Antibiotics must cover Staphylococcus aureus, and MRSA. Give:   - Co-trimoxazole 960mg PO twice a day   If co-trimoxazole is not available, give one of:   - Amoxicillin/clavulanic acid 500/125 mg PO three times a day - *or* Clarithromycin 500 mg PO twice a day - *or* Metronidazole 400mg PO three times a day   If IV antibiotics are needed, give   - a third-generation cephalosporin (e.g. ceftriaxone 2g IV twice a day)   If MRSA cannot be excluded, add one of:   - Clindamycin 600mg IV four times a day or - *or* a fluoroquinolone | Continue for 48 hours after symptoms have resolved |
| Orbital cellulitis | Antibiotics must cover Staphylococcus aureus and anaerobes. Give:   - Ceftriaxone 2g IV twice a day - *and* Metronidazole 500mg IV three times a day (PO acceptable if IV not available)   If MRSA cannot be excluded add one of:   - Co-trimoxazole 960mg PO twice a day PO - *or* Clindamycin 600mg IV four times a day - *or* Linezolid 600 mg PO twice a day | Minimum 2 weeks |
| Infectious keratitis | Moxifloxacin 0.5% or ciprofloxacin 0.3% eye drops: every 5-15 minutes for the first hour, then hourly for 48 hours,then hourly during the day until day 5, tapering thereafter according to clinical response.  If there is a strong risk of fungal keratitis, add Natamycin 5% drops (same frequency as fluoroquinolone, alternating between the two treatments every half hour). | If there is a poor response to treatment by day 5, consider treatment with cefuroxime 5% and gentamicin 1.5% in combination, 1 drop every hour during the first 48 hours, then every 2 hours until day 5. |

### Production of antibiotic eye drops from parenteral antibiotic preparations

The safe production of antibiotic eye drops requires adequate preparation and equipment, to avoid bacterial contamination. It is outlined in a book by the WHO (<https://iris.who.int/handle/10665/67840>), and in a comprehensive article [14].

In minimal-resource contexts where commercially prepared eye drops are not available, gentamicin drops can be prepared from parenteral (IV) gentamicin vials as follows. The strength of the drops will be higher than commercially available drops (which are 0.3%) but will be safe to use.

Combine the following volumes of gentamicin and sterile sodium chloride in a sterile syringe. A single syringe must be prepared for each patient, labelled, stored in a refrigerator and used for no more than 24 hours. A new syringe should be prepared every 24 hours and stored in a refrigerator, and the old syringe discarded.

| Gentamicin (IV) concentration | Volume of gentamicin to draw up | Volume of 0.9% sodium chloride to draw up | Total final volume | Final concentration |
| --- | --- | --- | --- | --- |
| 40mg/ml (4%) | 1ml | 4ml | 5ml | 0.8% |
| 80mg/ml (8%) | 1ml | 9ml | 10ml | 0.8% |

Ciprofloxacin is more complicated and may precipitate if refrigerated. IV ciprofloxacin is available as 400mg in 200ml (0.2%), which is weaker than ciprofloxacin eye drops (0.3%), but could be used if ciprofloxacin eye drops are not available (dosing should be more frequent). An alternative would be to give oral ciprofloxacin 750mg PO twice a day - although the extent to which this penetrates the eye is unclear [15].

**Useful equipment**

- Bright torch (for reflexes), and blue light-emitting diode (LED) torch
- Handheld ophthalmoscope
- Visual acuity charts: Snellen’s illiterate E chart or the [Peek Acuity App](https://peekvision.org/solutions/peek-acuity/) (available on Android phones)
- Eyedrops
  - Antibiotics (see above)
  - Local anaesthetics
  - Cyclopentolate
- Where there is no local ophthalmologist, a good quality phone camera is useful to take picture for referrals
- Slit lamps are a valuable aid to those trained to use them, as are ultrasound and x-ray

**Eye shields**

The aim of the eye shield is to avoid pressure on the eye: it can be improvised with a cone made of cardboard, or a plastic cup [9]. Do *not* apply an eye pad, which puts pressure on the eye. Instructions for how to make an eye shield have been published [16].


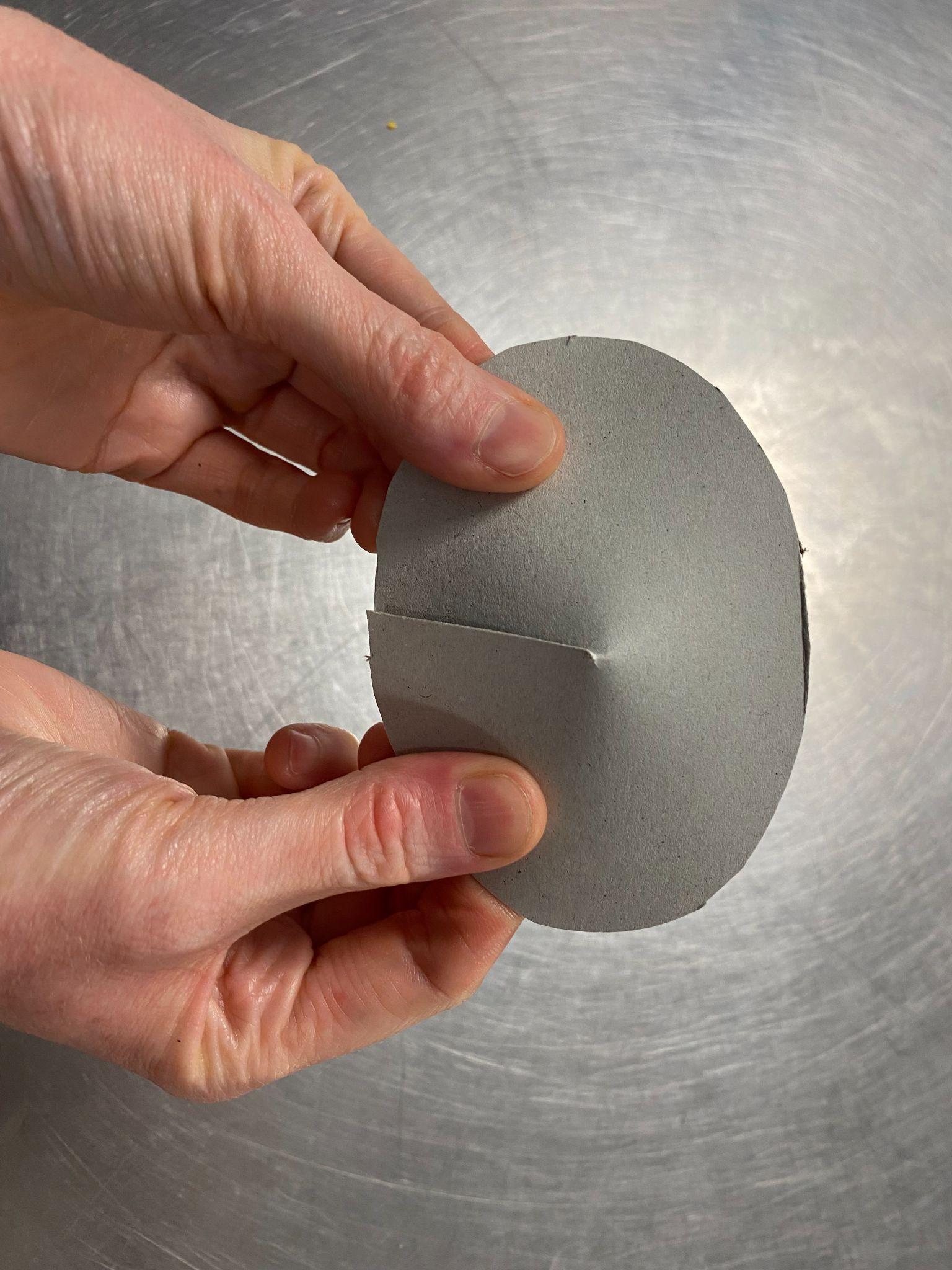

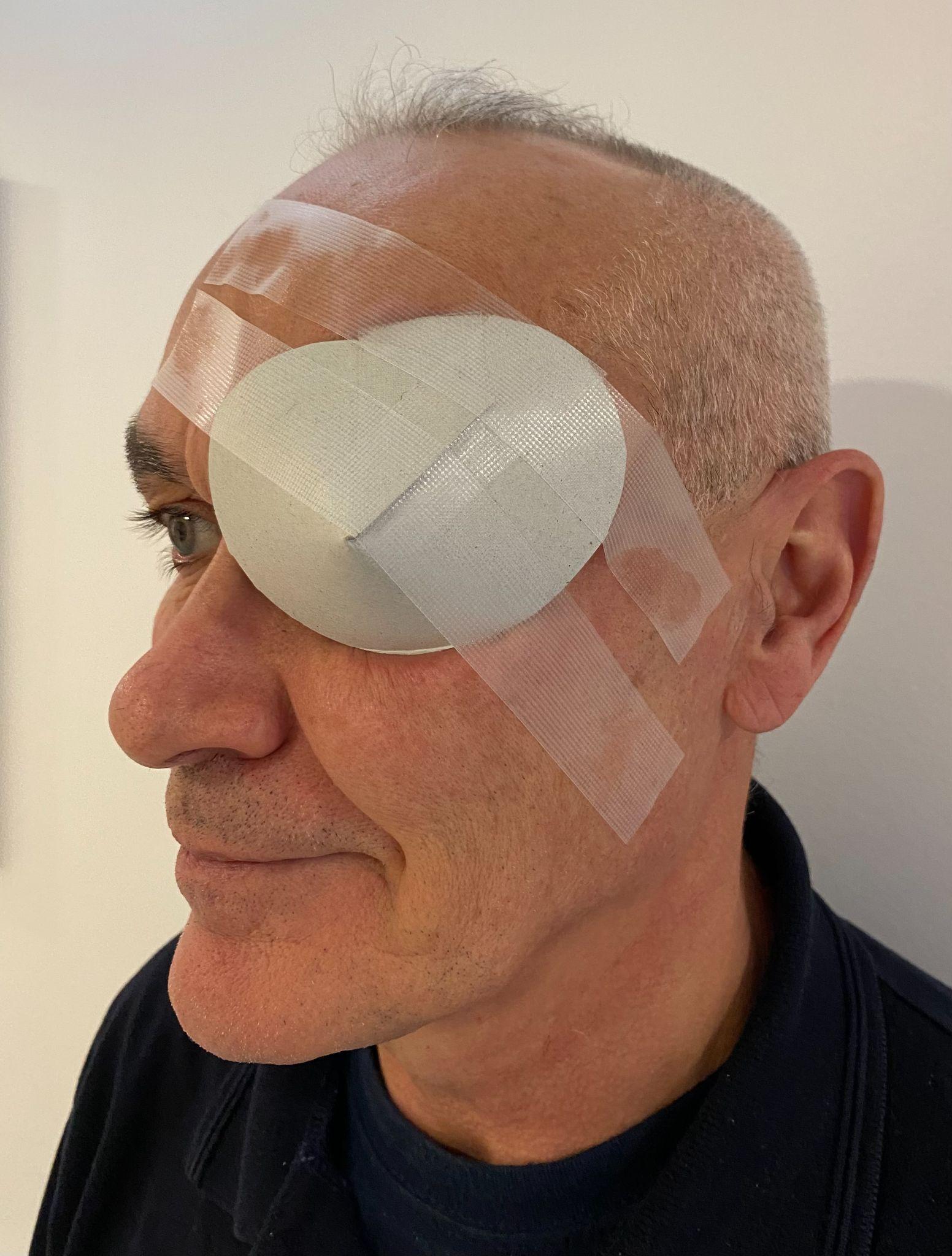


***Figure: improvised eye shield.***

Photo: Emma Butterfield, East Anglian Air Ambulance. Used with permission.

***Referral and transport***

- Ensure no pressure is put on the eye: do NOT pad the eye.
- Instead, put a protective shield over the eye: use a plastic eye shield, or a Styrofoam cup, taped to the face. Prevent the patient from touching it.
- Do not remove protruding foreign bodies unless there is a risk the patient will do so.
- Do not suture the eye.

### Glossary of ophthalmic terms

| **Term** | **Definition** |
| --- | --- |
| **Afferent pupillary defect (RAPD)** | A sign of optic nerve dysfunction, where one eye shows a reduced pupillary constriction to light; detected by the swinging flashlight test. |
| **Anterior chamber** | The fluid-filled space between the cornea and iris, containing aqueous humour. |
| **Aqueous humour** | The clear fluid produced by the ciliary body that fills the anterior and posterior chambers of the eye. |
| **Binocular indirect ophthalmoscopy** | Technique using a head-mounted light source and handheld lens to examine the retina; provides a wide field view. |
| **Blepharitis** | Inflammation of the eyelid margins, often chronic; not directly described but relevant to lid disorders. |
| **Blast injury** | Damage caused by high-pressure waves from explosions; in ocular trauma, can cause open or closed globe injuries. |
| **Blunt ocular trauma** | Injury from non-penetrating impact (e.g. fist, ball, stick) that may cause contusions, hyphema, or retinal detachment. |
| **Canthotomy (Lateral)** | Emergency surgical procedure to relieve orbital pressure by cutting the lateral canthal tendon; indicated in orbital compartment syndrome. |
| **Chemical eye injury** | Corneal and conjunctival damage from exposure to acids or alkalis; requires immediate irrigation to halt ongoing tissue destruction. |
| **Closed globe injury** | Eye trauma in which the outer wall (cornea and sclera) remains intact; includes contusions and lamellar lacerations. |
| **Conjunctiva** | The thin, transparent mucous membrane covering the sclera and inside of eyelids. |
| **Cornea** | The transparent front part of the eye covering the iris and pupil; key structure for refraction. |
| **Corneal abrasion** | Superficial defect or scratch of the corneal epithelium, usually causing pain, tearing, and photophobia. |
| **Corneal foreign body** | A particle embedded in, but not penetrating through, the cornea; requires careful removal to avoid infection or scarring. |
| **Cycloplegic** | A drug (e.g. cyclopentolate) that paralyses the ciliary muscle, reducing pain from ciliary spasm and preventing synechiae. |
| **Endophthalmitis** | Severe, sight-threatening infection involving the vitreous and/or aqueous humour, often after open globe injury or surgery. |
| **Enophthalmos** | Posterior displacement (sinking) of the eyeball within the orbit, often due to orbital fracture or volume loss. |
| **Evisceration** | Removal of intraocular contents, leaving the scleral shell intact; not performed in the field for trauma. |
| **Extraocular muscles** | Six muscles controlling eye movement; entrapment or injury can cause diplopia or restricted motility. |
| **Fluorescein staining** | Diagnostic technique using dye to highlight corneal epithelial defects or leaks (Seidel test). |
| **Globe rupture** | Full-thickness injury to the eye wall caused by blunt trauma; surgical emergency. |
| **Hyphaema** | Accumulation of blood in the anterior chamber, often due to blunt trauma; may raise intraocular pressure. |
| **Hypopyon** | Layer of white blood cells (pus) in the anterior chamber, typically signifying severe inflammation or infection. |
| **Intraocular foreign body (IOFB)** | Object lodged within the eye following penetrating injury; may be metallic or organic; requires urgent ophthalmic review. |
| **Intraocular pressure (IOP)** | Pressure within the eye (normal 10–21 mmHg); raised IOP can damage the optic nerve (glaucoma). |
| **Irrigation** | Flushing the eye with fluid (e.g. saline or clean water) to remove chemicals or debris. |
| **Lamellar laceration** | Partial-thickness wound of the cornea or sclera; the globe remains closed. |
| **Lateral canthal tendon** | Fibrous structure anchoring the eyelids laterally; divided during canthotomy to relieve orbital pressure. |
| **Open globe injury** | Full-thickness wound of the cornea or sclera caused by blunt rupture or sharp laceration; ophthalmic emergency. |
| **Orbital cellulitis** | Infection posterior to the orbital septum causing proptosis, ophthalmoplegia, and pain; requires IV antibiotics. |
| **Orbital compartment syndrome (OCS)** | Acute rise in orbital pressure compromising optic nerve perfusion; sight-threatening within 60–90 minutes. |
| **Orbital fracture** | Break in one or more bones of the orbit; may cause muscle entrapment or infraorbital nerve injury. |
| **Pupil** | Central aperture of the iris that regulates light entry into the eye. |
| **Red reflex** | Orange-red reflection from the retina when illuminated; loss suggests media opacity (e.g. hyphema, cataract). |
| **Retinal detachment** | Separation of the neurosensory retina from the underlying retinal pigment epithelium, causing visual field loss. |
| **Rupture (globe)** | Full-thickness break due to blunt force; often posterior; associated with poor prognosis. |
| **Sclera** | Dense white outer coat of the eyeball providing structure and protection. |
| **Seidel test** | Diagnostic test using fluorescein to detect aqueous humour leakage from corneal perforation (positive = open globe). |
| **Vitreous haemorrhage** | Bleeding into the vitreous cavity, often causing floaters or loss of vision after trauma. |
| **Zone of injury (ocular trauma classification)** | System to describe location of open-globe injuries: Zone 1 (cornea), Zone 2 (anterior sclera), Zone 3 (posterior sclera). |
